# Recombined humanized endostatin (Endostar) combined with chemotherapy for advanced bone and soft tissue sarcomas in stage IV

**DOI:** 10.18632/oncotarget.13545

**Published:** 2016-11-24

**Authors:** Peipei Xing, Jin Zhang, Zhao Yan, Gang Zhao, Xubin Li, Guowen Wang, Yun Yang, Jun Zhao, Ruwei Xing, Sheng Teng, Yulin Ma, Zhichao Liao, Zhiwu Ren, Chao Zhang, Xiuxin Han, Wei Zhang, Kexin Chen, Ping Wang, Jilong Yang

**Affiliations:** ^1^ Department of Bone and Soft Tissue Tumor, Tianjin Medical University Cancer Institute & Hospital, Tianjin 300060, People's Republic of China; ^2^ National Clinical Research Center of Cancer, Tianjin Medical University Cancer Institute & Hospital, Tianjin 300060, People's Republic of China; ^3^ Pharmacological Research Center, Tianjin Medical University Cancer Institute & Hospital, Tianjin 300060, People's Republic of China; ^4^ Department of Pathology, Tianjin Medical University Cancer Institute & Hospital, Tianjin 300060, People's Republic of China; ^5^ Department of Radiology, Tianjin Medical University Cancer Institute & Hospital, Tianjin 300060, People's Republic of China; ^6^ Department of Cancer Biology, Wake Forest Baptist Comprehensive Cancer Center, Winston-Salem, North Carolina 27157, USA; ^7^ Department of Epidemiology and Biostatistics, Tianjin Medical University Cancer Institute & Hospital, Tianjin 300060, People's Republic of China; ^8^ Department of Radiation Oncology, Tianjin Medical University Cancer Institute & Hospital, Tianjin 300060, People's Republic of China

**Keywords:** sarcoma, endostar, chemotherapy, progression free survival, clinical benefit response

## Abstract

**Purpose:**

This retrospective case-series study evaluated efficacy and safety of Endostar combined with chemotherapy in the treatment of advanced bone and soft tissue sarcomas in stage IV.

**Materials and Methods:**

Forty-seven patients diagnosed with stage IV bone and soft tissue sarcomas and treated with chemotherapy in Tianjin Medical University Cancer Institute & Hospital were reviewed. Of these patients, 23 patients were treated with Endostar plus chemotherapy (designated as combined group), and 24 patients received only chemotherapy (designated as control group). Progression-free survival (PFS), overall survival (OS), objective response rate (ORR) and clinical benefit response (CBR) were analyzed to find the difference between these two groups with the purpose to investigate the role of Endostar in metastatic sarcomas.

**Results:**

Endostar combined with chemotherapy had significantly increased PFS. In the combined group and control groups, the median PFS (8.6 months versus 4.4 months) and the CBR (47.8% versus 16.7%) showed significant difference (*P* = 0.032), while the median overall survival (11.7 months versus 10.6 months, *P* = 0.658) and the ORR (17.4% versus 8.3%, *P* = 0.167) showed no significant difference. The common grade 3-4 side effects for both groups were myelosuppression and transient elevation of transaminases.

**Conclusion:**

Endostar combined with chemotherapy had significant activity to increase the PFS and improve CBR in patients with advanced sarcomas, with tolerable side effects.

## INTRODUCTION

Sarcoma, a heterogeneous group of malignancies that arise from mesenchymal tissue, is mainly divided into two categories: soft tissue sarcoma and bone sarcoma. Sarcomas account for approximately 1% of all adult malignancies and 15% of pediatric malignancies in the United States, where an annual increase of about 12020 cases of soft tissue sarcoma patients in 2014, among which 4720 cases were died of the disease [1]. Soft tissue sarcomas (STS) include more than 50 different subtypes: the most common pleomorphic undifferentiated sarcoma (25% ~ 35%), followed by liposarcoma (25% ~ 30%), leiomyosarcoma (12%), synovial sarcoma (10%) and malignant peripheral nerve sheath tumor (6%) [2]. Despite the different pathological types, the 10% patients with high-grade STS already have metastasis when newly diagnosed [3]. Osteosarcoma is the most common primary malignant tumor, common in children and young adolescents and the osteosarcoma was newly diagnosed with the distant metastasis rate of 20% ~ 40% [3, 4]. The lung is the most common site of metastasis in patients with bone and soft tissue sarcomas.

Patients with metastatic soft tissue sarcoma had a poor treatment effect, with the median survival time of 12 months, what's worse, the 5 year survival rate was lower than 10% in some large-scale studies [5, 6]. Similarly, patients with advanced osteosarcoma who had pulmonary metastases also had a poor prognosis, with the overall survival rate of 0%~50% [7]. The classic chemotherapy such as ifosfamide, doxorubicine, methotrexate, cisplatin, dacarbazine, gemcitabine and docetaxel is unresectable for cure, although chemotherapy plays a major role in the treatment of advanced soft tissue sarcoma and bone sarcoma [8, 9]. Beside, combination or dose-dense regimens have largely failed to improves the response rates as what the oncologist`s have expected [10, 11]. Researchers have also shown that long-term using of cytotoxic drugs increased the risk of toxicity in patients, such as cumulative dose and dose intensity of doxorubicin cause cardiomyopathy and an associated mortality risk [12, 13]. Therefore, there is a need for new combination chemotherapies or other methods for the treatment of advanced sarcoma.

Angiogenesis is a key factor for tumor growth and metastasis both in cancer and sarcoma. Thus, anti-angiogenesis therapy has become a new field of tumor therapy [14]. Endostatin is the strongest endogenous angiogenesis inhibitor, which inhibits vascular endothelial growth factor (VEGF) expression and then inhibits tumor angiogenesis [15]. Endostar, is a novel recombinant human endostatin, with advantages of long half-life, stable and low cost [16]. Recently, a study of Endostar combined with chemotherapy in the treatment of advanced soft tissue sarcoma indicated resulted in a higher clinical benefit response (CBR) and longer progression-free survival (PFS), with tolerable side effects [17]. However this study included the patients with stage ΠB-IV soft tissue sarcomas and did not include specific pathologic information. Thus the present study was carried to compare the efficacy and safety of endostar combined with chemotherapy versus chemotherapy alone in stage IV patients with bone and soft tissue sarcomas. Our data validated the efficacy and safety of Endostar combined with chemotherapy in the treatment of patients with stage IV bone and soft tissue sarcomas.

## RESULTS

### Patients and treatment

Between June 2008 and December 2015, a total of 47 patients diagnosed with advanced bone and soft tissue sarcomas in stage IV were treated with chemotherapy. 23 patients with sarcomas in stage IV were treated with Endostar combined chemotherapy. While in the same period, 24 metastatic sarcoma patients received only chemotherapy because of the poor economic status and/or the apprehensions about Endostar. We reviewed these 47 cases with the purpose to investigate the role of Endostar combined with chemotherapy. We divided them into two groups, the patients received Endostar combined with chemotherapy (named as combined group) and the patients treated with only chemotherapy (named as control group).

Patient base line characteristics are described in Table 1, and the characteristics of two groups were equilibrium (*P* > 0.05). All patients had stage IV disease according to AJCC (American Joint Committee on Cancer). The pathological types of the two groups were mainly osteosarcoma, synovial sarcoma and undifferentiated pleomorphic sarcoma (UPS), less frequent types such as leiomyosarcoma and malignant peripheral nerve sheath tumor (MPNST). Among the 47 patients, 5 patients were newly diagnosed sarcomas in stage IV (3 patients in combined group and 2 patients in control group). The other 42 patients were relapsed sarcomas in stage IV after the treatment of localized disease (20 patients in combined group and 22 patients in control group). The majority of the patients had received wide resection or radical resection surgery, while small part of the patients received radiotherapy.

**Table 1:** Baseline characteristics of combined and control groups

	No. (%) of patients		
Variable	combined group (n = 23)	control group (n = 24)	*P*
**Age (years)**			
Mean (range)	36 years (12-68ys)	34 years (15-70ys)	0.650
**Sex**			
Male	13 (56.5)	18 (75.0)	0.227
Female	10 (43.5)	6 (25.0)	
**Histology**			
osteosarcoma	8 (34.8)	12 (50.0)	0.665
synovial sarcoma	4 (17.4)	6 (25.0)	
UPS	4 (17.4)	1 (4.2)	
leiomyosarcoma	1 (4.3)	1 (4.2)	
chondrosarcoma	1 (4.3)	1 (4.2)	
other types	5 (21.7)	3 (12.5)	
**KPS**			
70	7 (30.5)	3 (12.5)	0.344
80	5 (21.7)	7 (29.2)	
90	11 (47.8)	14 (58.3)	
**Site**			
femur	6 (26.1)	3 (12.5)	0.359
tibia	2 (8.7)	6 (25.0)	
humerus	1 (4.3)	3 (12.5)	
thigh / hip	6 (26.1)	3 (12.5)	
leg / foot	4 (17.4)	2 (8.3)	
shoulder	2 (8.7)	1 (4.2)	
abdominopelvic	1 (4.3)	2 (8.3)	
other sites	1 (4.3)	4 (16.7)	
**Patient (stage IV) types**			
newly diagnosed	3 (13.0)	2 (8.3)	0.666
relapsed	20 (87.0)	22 (91.7)	
**Previous surgery**			
excision biopsy	3 (13.0)	2 (8.3)	0.610
wide resection	11 (47.8)	15 (62.5)	
radical resection	9 (39.2)	7 (29.2)	
**Previous radiotherapy**			
yes	6 (26.1)	2 (8.3)	0.137
no	17 (73.9)	22 (91.7)	
**Chemotherapy regimen after metastasis**			
AI	6 (26.1)	6 (25.0)	0.067
MAID	1 (4.3)	6 (25.0)	
GT	12 (52.3)	2 (8.3)	
T10	3 (13.0)	10 (41.7)	
PEM plus Cisplatin	1 (4.3)	0 (0)	

Abbreviations: KPS, Karnofsly's Performance Status; UPS, undifferentiated pleomorphic sarcoma.

P value from χ2 and t-test for categorical and continuous covariates, respectively.

The chemotherapy regimens of patients after distant metastasis were mainly consisted of AI, GT, MAID, CYVADIC, T10, and Pemetrexed plus Cisplatin. The comparison of major chemotherapy regimen of patient in the combined group and the control group was showed in Table 1. The patient with osteosarcoma used T10 as the main chemotherapy method, while patients with soft tissue sarcomas primarily used AI or GT. In the combined group, the number of Endostar using cycles was at least two cycles.

### Efficacy

As shown in Table 2, the clinicopathological factors associated with survival were examined, and only Endostar combined with chemotherapy had significant activity to increase the PFS (χ2 = 4.612, *P* = 0.032), while other clinical pathological features and treatments had no significant impact on PFS. Relapsed patients after the treatment of localized disease and patients received wide resection or radical resection surgery had a significant longer overall survival (OS), while Endostar combined with chemotherapy had no significant impact on OS (χ2 = 0.196, *P* = 0.658). Cox regression survival analysis found no independent prognostic factor for OS.

**Table 2:** Prognostic role of Endostar in 47 patients with advanced sarcomas

	PFS		OS	
Variable	*χ2*	*P*	*χ2*	*P*
**Age (≥ 40 year, < 40 year)**	1.174	0.279	0.663	0.416
**Sex**	1.403	0.236	0.003	0.958
**Histology**	7.790	0.168	1.739	0.884
**KPS**	3.609	0.165	2.779	0.249
**Site**	6.329	0.502	4.909	0.671
**Relapsed**	3.591	0.058	6.007	0.014
**Previous surgery**	3.967	0.138	6.480	0.039
**Previous radiotherapy**	3.140	0.076	2.895	0.089
**Chemotherapy regimen**				
AI	0.832	0.362	2.147	0.143
MAID	0.274	0.660	0.303	0.582
GT	0.145	0.704	1.246	0.264
T10	1.131	0.288	1.316	0.251
**Endostar**	4.612	**0.032**	0.196	0.658

**Figure 1:**
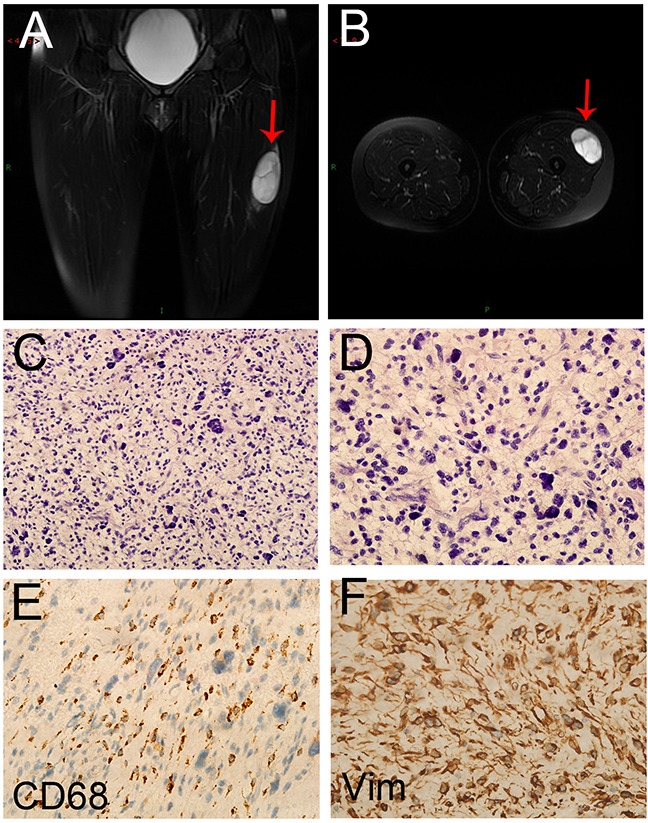
The image and pathological data of the primary lesion in the CR patient **A,B**. The preoperative MRI of the patient revealed a 63×45×27 mm mass (arrow) in the left thigh. (C) HE staining of the primary lesion (20×). (D) HE staining of the primary lesion (40×). **C,D**. The postoperative pathological diagnosis showed the result of undifferentiated pleomorphic sarcoma (UPS). **E**. CD68 protein expression in UPS (40×). **F**. Vimentin protein expression in UPS (40×).

**Figure 2:**
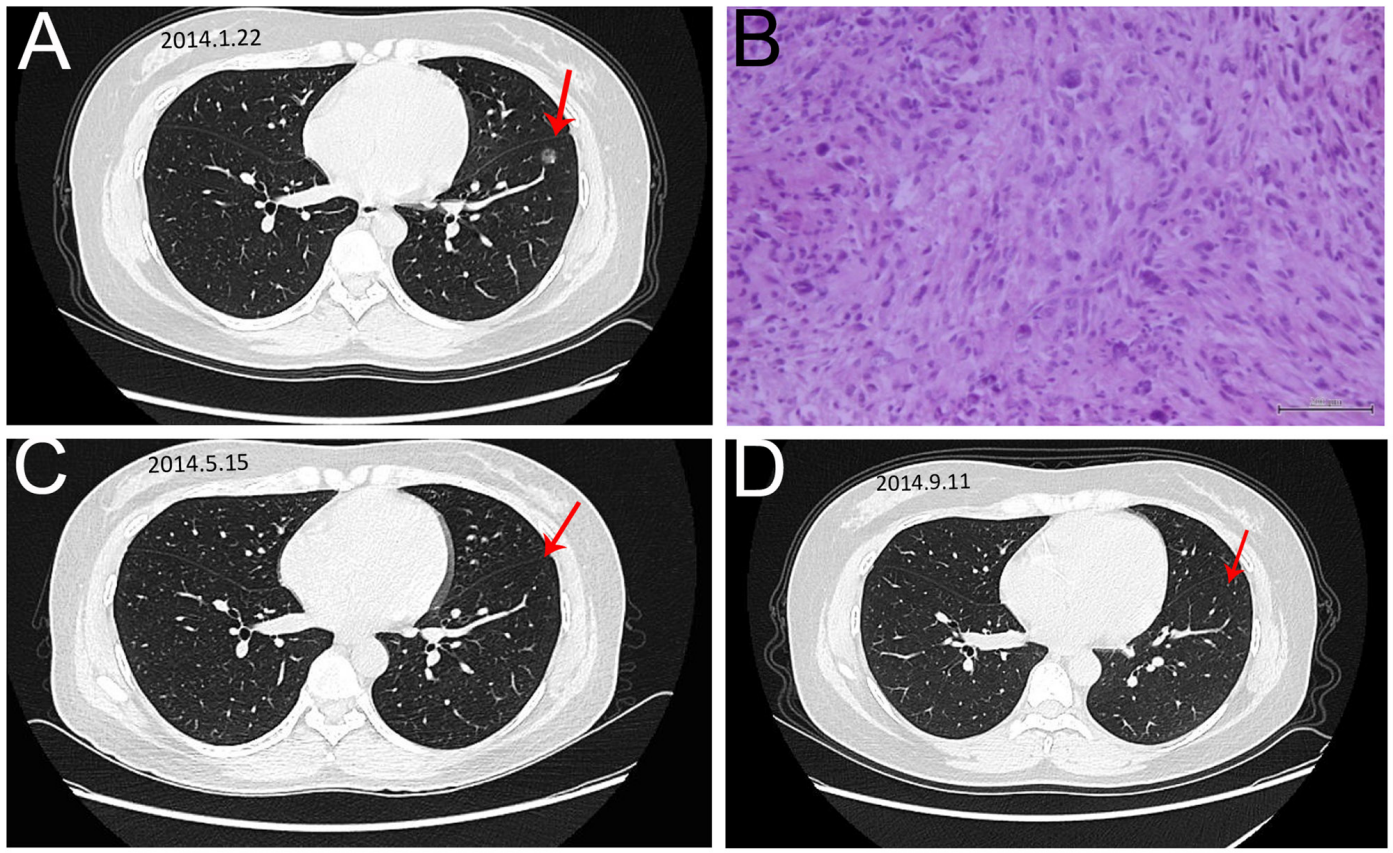
The image and pathological data of the left lung metastase in the CR patient **A**. The chest CT showed there was a 1.2 × 1.2 cm nodule (arrow) in the left lung. **B**. HE staining of the Lung metastasis (20×). The result of needle aspiration biopsy of pulmonary nodule was UPS and the patient was diagnosed with lung metastasis. **C,D**. The repeated chest CT showed that the disappearance of lung metastase in 2 time intervals.

Important indexes of the two groups that reflect disease control were shown in Table 3. There was one complete response (CR) case in combined group, while the control group had no CR case. This female patient was first diagnosed with soft tissue sarcoma in the left thigh in June 2013 (Figure 1A–1B). After the wide resection of tumor in the left thigh, the patient was diagnosed as undifferentiated pleomorphic sarcoma (UPS) with positive CD 68 and Vimentin expression (Figure 1C–1F). The adjuvant treatment after surgery include 50 Gy radiotherapy, 3 cycles chemotherapy with AI regiments and 2 cycles of CYVADIC regiments. 2 months after the primary treatment, the chest CT showed there was a 1.2 × 1.2 cm nodule in the left lung (Figure 2A), and the result of needle aspiration biopsy of pulmonary nodule was confirmed as the metastasis of UPS (Figure 2B). Due to the Doxorubicin resistance, we chose GT regiment. After treated with 8 cycles of Endostar combined with 6 cycles of chemotherapy of GT, the patient was evaluated as CR with the left lung metastatic tumor disappearing (Figure 2C–2D). The patient is currently maintained Endostar treatment alone, without progress in the disease (Supplementary Figure S1A).

**Table 3:** End points that reflect disease control in two groups

End Point	combined group(n = 23)	control group(n = 24)	χ^2^	*P*
**PFS, months**	8.6	4.4	4.612	**0.032**
**OS, months**	11.7	10.6	0.196	0.658
**CR**	1 (4.3)	0 (0)	5.805	0.086
**PR**	3 (13.0)	2 (8.3)		
**SD**	7 (30.4)	2 (8.3)		
**PD**	12 (52.3)	20 (83.4)		
**CBR**	11 (47.8)	4 (16.7)	4.612	**0.032**
**ORR**	4 (17.4)	2 (8.3)	1.913	0.167

Abbreviations: PFS, progression-free survival; OS, overall survival; CR, complete remission; PR, partial remission; SD, stable disease; PD, progressive disease; CBR, clinical benefit rate; ORR, objective response rate.

**Figure 3:**
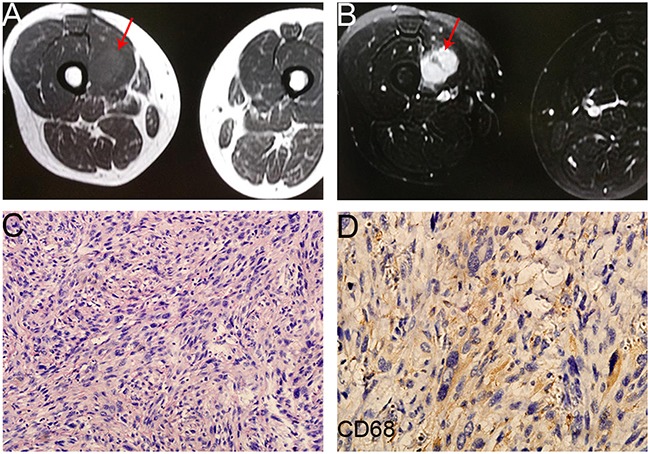
The image and pathological data of the primary lesion (the right thigh) in one PR patient **A,B**. The preoperative MRI of the patient (axv FSE T1 + axial fs FSE T2) showed a mass (arrow) on the right thigh. **C**. HE staining of the primary lesion (20×) showed the diagnosis of UPS. **D**. CD68 protein expression in UPS tissue sample (40×).

**Figure 4:**
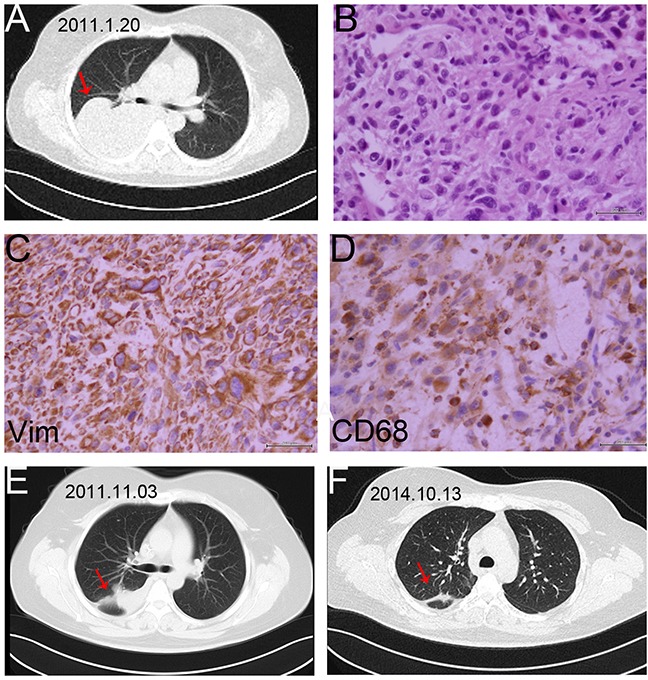
The image and pathological data of the right lung metastasis in one PR patient **A**. The chest CT showed there was an 11 × 11cm tumor (arrow) in the right lung. **B**. HE staining of the lung metastasis (40×). The pathological result of needle aspiration biopsy of pulmonary lesion was UPS, and the patient was diagnosed with lung metastasis. **C**. Vimentin protein expression in UPS tissue sample of lung (40×). **D**. CD68 protein expression in UPS tissue sample of lung (40×). **E,F**. The repeated chest CT showed the result of PR with the volumes of right lung metastases tumor reducing evidently in 2 time intervals.

Partial response (PR) cases were more frequently observed in the combined group compared with the control group [13.0% (3/23) versus 8.3% (2/24)]. The imaging and pathological data of one case of PRs were shown as follows. The female patient was first diagnosed with soft tissue sarcoma in the right thigh (Figure 3A–3B). The pathological diagnosis after wide resection was UPS with positive CD68 expression (Figure 3C–3D). The adjuvant treatment after surgery include 50 Gy radiotherapy, 3 cycles chemotherapy with AI regiments and 3 cycles of CYVADIC regiments. 2 years after the primary treatment, the chest CT showed there was a 11 × 11cm mass in the right lung (Figure 4A), and the result of needle aspiration biopsy of pulmonary lesion was UPS with positive CD68 and Vimentin (Figure 4B–4D). Due to the unacceptable toxicity of AI regiment, we chose GT chemotherapy. After treated with 8 cycles of Endostar combined with 6 cycles chemotherapy of GT, and continued Endostar alone for 4 cycles, the patient was evaluated as PR with the volumes of right lung metastases tumor was reduced evidently (Figure 4E–4F) (Supplementary Figure S1B).

As shown in Table 3, the total CBR in the combined group was over 65% greater than in the control group (47.8 % versus 16.7%, respectively; χ2 = 4.612, *P* = 0.032). The CBR reflects both the treatment response to drugs and the stable duration of the disease, was significantly higher in the combined group (Table 3). Even the objective response rate (ORR) in the combined group was higher than that of control group (17.4% versus 8.3%, respectively), there was no significantly difference between these two groups (χ2 = 1.913, *P* = 0.167) (Table 3).

**Figure 5:**
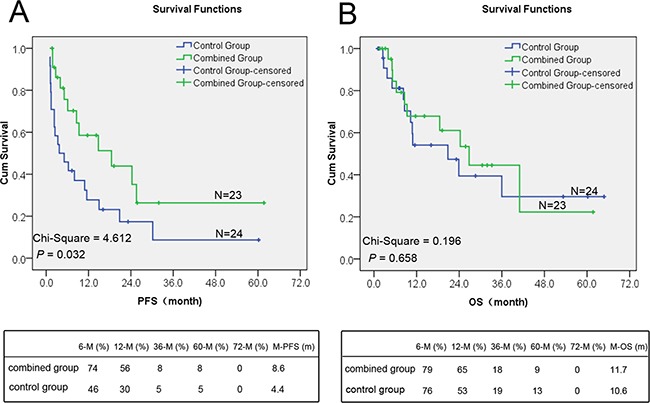
The difference of PFS and OS in the combined and control groups **A**. The difference of the median PFS between the control group and the combined group was statistically significant (*P* < 0.05). The PFS of combined group was significantly better than that of control group. **B**. The difference of the overall survival between the two groups was statistically insignificant (*P* > 0.05).

The final analysis showed that treatment with Endostar plus chemotherapy resulted in a better PFS compared with chemotherapy alone (χ2 = 4.612, *P* = 0.032) (Figure 5A). Endostar plus chemotherapy led to an improved median PFS compared with chemotherapy alone (8.6 months versus 4.4 months, *P* = 0.032). The PFS of 6,12,36 and 60 months in the combined group was 74%, 56%, 8%, and 8% respectively, while in the control group it was 46%, 30%,5%, and 5%, respectively (Figure 5A). The interim analysis of OS demonstrated an insignificantly difference in combined group compared with control group (median OS for combined group versus control group, 11.7 versus 10.6 months; χ2 = 0.196, *P* = 0.658) (Figure 5B). The one-year survival rate in the combined group and control group was 65% and 53%, respectively, while the five years survival rate was 9% and 13%, respectively (Figure 5B).

### Toxicity

The main adverse reactions, including nausea, vomiting, heart electrocardiogram abnormalities, were generally tolerable. The common grade 3-4 side effects were myelosuppression and transient elevation of transaminases. There was no statistically difference in side effects between both groups. There was neither 1 case termination of treatment, nor treatment-related death in all patients (Table 4).

**Table 4:** Most common grade 3 or 4 adverse events in the combined and control groups

Adverse Event	No. (%) Adverse Events by Treatment		*P*
	combined group	control group	
**myelosuppression**			
WBC decrease	8 (34.8)	7 (29.2)	0.760
neutrophils decrease	8 (34.8)	7 (29.2)	0.763
hemoglobin decrease	6 (26.1)	4 (16.7)	0.494
thrombocyte decrease	2 (8.7)	4 (16.7)	0.666
**transaminases rise**	2 (8.7)	1 (4.2)	0.609
**ECG abnormalities**	3 (13.0)	1 (4.2)	0.472

Abbreviations: WBC, white blood cell; ECG, electrocardiogram.

## DISCUSSION

Endostar, as a new recombinant human endostatin, is a multi-target tumor cell inhibitor [16]. Multiple studies showed that endostar not only directly suppressed the VEGF-stimulated proliferation and migration, but also suppressed the VEGF-induced VEGFR-2 expression and the activation of ERK, p38 MAPK, and AKT, and then inhibit tumor progression [16, 18]. Endostar combined with chemotherapy has achieved significant clinical benefits in a number of solid tumors such as lung cancer, breast cancer, gastrointestinal tumor, cervical cancer and neuroendocrine tumor [19–23]. Endostar has been granted by the State Food and Drug Administration of China (SFDA) in 2005 for the treatment of non-small-cell lung cancer (NSCLC).

Due to the high heterogeneity of pathological types of sarcoma, their sensitivities to chemotherapy are different, but overall, metastasized sarcoma has lower 5 year survival rate [24]. Thus, new therapeutic strategies for sarcoma are needed. The researches about the treatment of Endostar combined with chemotherapy of sarcoma have made some progresses. A retrospective case-series study in the patients with stage IIB-IV soft tissue sarcomas demonstrated Endostar combined with chemotherapy led to a higher CBR and longer PFS, without significant different in OS and ORR [17]. In addition, a study in newly diagnosed patients with osteosarcoma showed that Endostar combined with chemotherapy resulted in a higher 5-year event-free survival rate compared with control group (70% versus 56%, *P* = 0.043), and led to a lower metastasis rate [25]. However, there is still no typical study to investigate the role of Endostar in sarcoma patients in stage IV.

The present retrospective case-series study evaluated efficacy and safety of endostar combined with chemotherapy in the treatment of advanced sarcoma. The results show that Endostar combined with chemotherapy result in a higher CBR and a longer median PFS in the patients with advanced sarcoma (8.6 months versus 4.4 months), with tolerable toxicity, which are consistent with previous reports of endostar combined with chemotherapy in other solid tumors [26–28]. In our study, the PFS of the combined group is significantly longer than that of the control group. For the short-term therapeutic effects, evaluated by CBR and ORR, the CBR of the combined group was significantly higher than that of the control group, while the difference in ORR between the two groups was insignificant. These data suggest that chemotherapy combined with Endostar in treatment of advanced bone and soft tissue sarcomas could significantly improve the clinical benefit, and prolong progression free survival, while not increase adverse reactions compared with chemotherapy alone. More cases and longer follow-up in the setting of random trials are warranted to further validate these results.

However, there are still some issues about this novel finding. First of all, this study is retrospective one, but not randomized controlled trial. However, the patient base line characteristics of these two groups, such as the tumor type, site, KPS, surgery, radiotherapy, chemotherapy regiments, et al., are equilibrium, which make up the flaw of nonrandomized control. According to the present exciting results, randomized controlled study has been launched in our sarcoma center and would be better to investigate the role of endostar combined with chemotherapy. Secondly, because of the rarity of sarcomas, the sample in present study is small. It is hard to evaluate the effects of specific chemotherapy regiments or sarcoma types. So long-term randomized controlled study with more sarcoma patients, types and chemotherapy regiments should be performed to figure out the role of these factors. Thirdly, the follow up of present study is short because some of the patients are still alive. Long-term follow up are going on and we would supply new data when necessary. Although these flaws existed, our present study do provide a new treatment choice for the rarely sarcoma patients in stage IV. In the future, long-term randomized controlled study with more cases would benefit more sarcoma patients.

## MATERIALS AND METHODS

### Patients

In the retrospective case-series study, 47 cases of patients with diagnosis of metastatic bone or soft tissue sarcomas (stage IV) from the Tianjin Medical University Cancer Institute & Hospital between June 2008 and December 2015 were treated with chemotherapy and analyzed. 23 patients with sarcomas in stage IV treated with Endostar combined chemotherapy. While in the same period, 24 metastatic sarcoma patients received only chemotherapy because of the poor economic status and/or the apprehensions about Endostar. We reviewed these 47 cases with the purpose to investigate the role of Endostar combined with chemotherapy. We divided them into two groups, the patients received Endostar combined with chemotherapy (named as combined group) and the patients only treated with chemotherapy (named as control group).

### Treatment procedures

Endostar was given at a dose of 15mg once daily by intravenous infusion for 14 days combined with chemotherapy regimens, and 21 days was a cycle. The chemotherapy was administered according to follows: Ifosfamide 8-12 g/m^2^, Doxorubicine 75 mg/m^2^, Methotrexate 8-12 g/m^2^, Cisplatin 80-120 mg/m^2^, Dacarbazine 200-400 mg/m^2^, Gemcitabine 1000 mg/m^2^, Docetaxel 75 mg/ m^2^. They were used by single-agent or combination regimens according to National Comprehensive Cancer Network (NCCN) and the European Society for Medical Oncology (ESMO). The combined regimens included AI, MAID, GT, CYVADIC and T10 [29].

### Assessments

The Clinical benefit response was according to Response Evaluation Criteria in Solid Tumors (RECIST). (CBR) = (CR+PR+SD)/ total number of cases × 100%, and (ORR) = (CR+PR) / total number of cases × 100%. Safety evaluation included medical history and physical examination, measurement of blood pressure, blood routine, blood biochemistry, urine routine and urine protein. Adverse events were graded according to the Common Terminology Criteria for Adverse Events, version 3.0 [30]. Evaluation of adverse events was conducted after 1 cycle, and the efficacy evaluation was carried out at least after 2 cycles. Patients were treated until tumor progression or unacceptable toxicity.

The information of PFS, OS, ORR and CBR were analyzed in both two groups. The indexes such as CR, PR, or stable disease (SD) which reflects the number and percentage of patients who achieved RECIST were also reviewed. The PFS was the time from first treatment when diagnosed with stage IV to disease progression, while OS was the time from first treatment when diagnosed with stage IV to death.

The patients were followed up to death or 20 March, 2016 by means of reviewing or on the phone.

### Statistical analyses

Correlations between combined group and control group in clinicopathological variables were analyzed using the Chi-square test. The primary objective of this study was PFS and OS, which were estimated using Kaplan-Meier, and the comparison of the survival rates of the two groups was log-rank test. The Cox regression survival analysis was used to do the multivariate analysis. The statistical methodology for ORR and CBR was Fisher's exact test. All of statistical tests were two-sided, with the significance level of 0.05.

## SUPPLEMENTARY FIGURE


