# Supplementary material for: Recombined humanized endostatin (Endostar) combined with chemotherapy for advanced bone and soft tissue sarcomas in stage IV
Source: Oncotarget. 2016 Nov 24;8(22):36716–27. doi: 10.18632/oncotarget.13545 (PMC5482691; doi:10.18632/oncotarget.13545)
Supplement: Supplementary file 1 [file oncotarget-08-36716-s001.pdf]

Recombined humanized endostatin (Endostar) combined with chemotherapy for advanced bone and soft tissue sarcomas in stage IV

SUPPLEMENTARY FIGURE

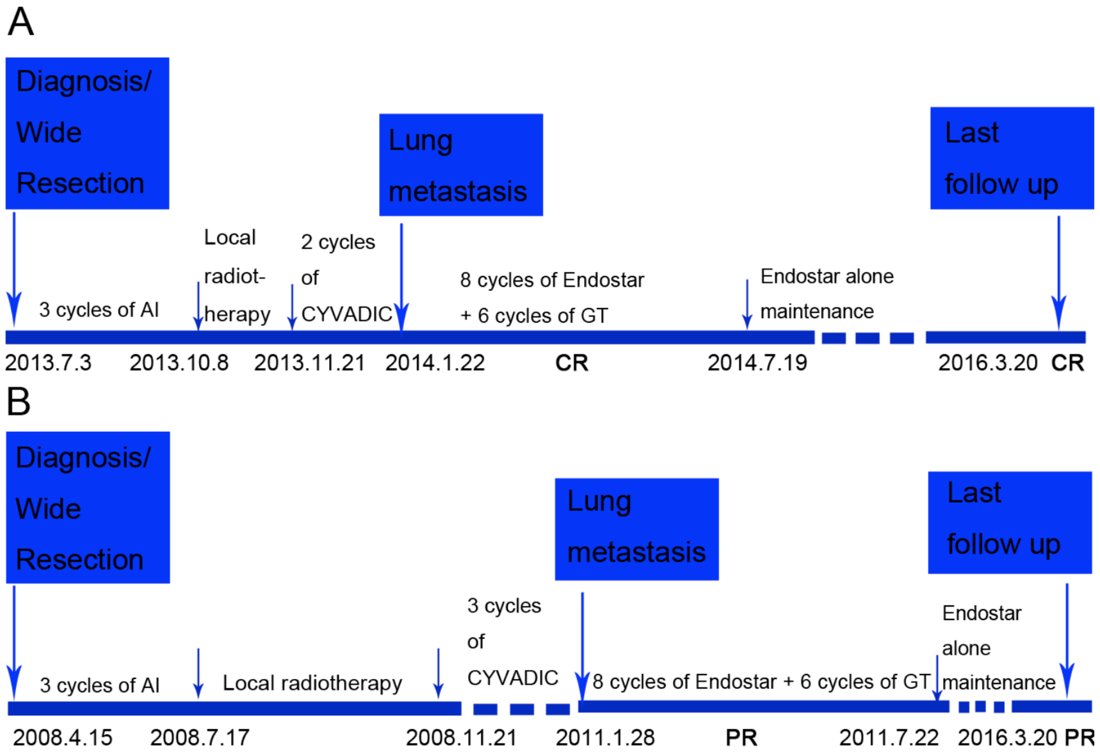

Supplementary Figure S1: The timeline of two typical cases. A. The timeline of diagnosis and treatment in CR patient. **B.** The timeline of diagnosis and treatment in PR patient.
